# Surgery for degenerative cervical spine disease in Finland, 1999–2015

**DOI:** 10.1007/s00701-019-03958-6

**Published:** 2019-06-01

**Authors:** Anna Kotkansalo, Ville Leinonen, Merja Korajoki, Jyrki Salmenkivi, Katariina Korhonen, Antti Malmivaara

**Affiliations:** 1 Division of Clinical Neurosciences, Department of Neurosurgery, Turku University Hospital, PB 52, 20521 Turku, Finland; 2 Faculty of Medicine, Department of Clinical Medicine, University of Turku, Turku, Finland; 3 Centre for Health and Social Economics, National Institute for Health and Welfare, Helsinki, Finland; 4 Unit of Clinical Neuroscience, Neurosurgery, University of Oulu and Medical Research Center, Oulu University Hospital, Oulu, Finland; 5 Institute of Clinical Medicine – Neurosurgery, University of Eastern Finland and Department of Neurosurgery, Kuopio University Hospital, Kuopio, Finland; 6 Department of Orthopedics, Helsinki University Hospital, Helsinki, Finland; 7 Welfare District of Forssa, Forssa, Finland; 8 Orton Orthopaedic Hospital, Helsinki, Finland

**Keywords:** Degenerative cervical spine, Incidence, Nationwide register study, Regional differences, Surgery, Trends

## Abstract

**Background:**

The incidence of surgery for degenerative cervical spine disease (DCSD) has risen by almost 150% in the USA in the last three decades and stabilized at slightly over 70 operations/100,000 people. There has been significant regional variation in the operation incidences. We aim to assess the diagnosis-based, age-adjusted trends in the operation incidences and the regional variation in Finland between 1999 and 2015.

**Methods:**

Data from the Finnish Hospital Discharge Register (FHDR), the Cause of Death Register, and the registers of the Social Insurance Institution were combined to analyze all the primary operations for DCSD or rheumatoid atlanto-axial subluxation (rAAS). Combinations of the operative and the diagnosis codes were used to classify the patients into five diagnostic groups.

**Results:**

A total of 19,701 primary operations were included. The age-adjusted operation incidence rose from 21.0 to 36.5/100,000 people between 1999 and 2013 and plateaued thereafter. The incidence of surgery for radiculopathy increased from 13.1 to 23.3 operations/100,000 people, and the incidence of surgery for DCM increased from 5.8 to 7.0 operations/100,000 people. The rise was especially pronounced in surgery for foraminal stenosis, which increased from 5.3 to 12.4 operations/100,000 people. Of the five diagnostic groups, only operations for rAAS declined. Operations increased especially in the 40- to 65-year-old age group. The overall operation incidences varied from 18.3 to 43.1 operations/100,000 people between the university hospitals.

**Conclusions:**

The age-adjusted incidence of surgery for DCSD has risen in Finland by 76%, but the rise has plateaued. Surgery for radiculopathy, especially for foraminal stenosis, increased more steeply than surgery for degenerative medullopathy, with vast regional differences in the operation incidences.

**Electronic supplementary material:**

The online version of this article (10.1007/s00701-019-03958-6) contains supplementary material, which is available to authorized users.

## Introduction

Data on the incidence or the prevalence of radiculopathy or especially myelopathy is scarce. Between 83.2 and 179/100,000 people have been diagnosed annually with radiculopathy [29, 33] and in a door-to-door survey, radiculopathy was found in 350 per 100,000 people [31]. The prevalence of spondylotic myelopathy (CSM) is not known [7, 12, 41], but in Taiwan and in the United States (US), 4.04 people per 100,000 person-years [40] and 7.88 per 100,000 people [16], respectively, were hospitalized for CSM. A radiological study of cervical MRI scans found radiological signs of myelomalacia unrelated to trauma or previous surgery in 4.2% of patients age 18 or older [41].

The benefit of surgery over conservative treatment for degenerative cervical spine disease (DCSD) has not been unequivocally demonstrated [6, 15, 38, 39]. Nevertheless, the estimated rate of surgery for degenerative cervical symptoms has risen steadily from the 1970s onwards until the last decade in the US [3, 9, 18, 22, 24, 26] and stabilized at between 70 and 80 operations/100,000 people [18] (a summary of the previous literature is provided in the Supplementary Table S1). A similar rise in the operation rates has been recently demonstrated in Norway as well [14]. However, systematic longitudinal nationwide incidences for the different diagnostic entities have not been reported.

We aim to assess the trends and regional variation in the incidence of operations for degenerative and rheumatoid cervical spine disease independent of the change in the population age and sex distribution. The analysis includes every primary operation performed between 1999 and 2015 in both public and the private hospitals in Finland, which has a regionally organized hierarchical, tax-funded health care system accessible to all residents.

## Materials and methods

### Study design and data sources

The PERFECT (PERFormance, Effectiveness, and Cost of Treatment episodes) Cervical Spine database was created by retrospectively combining data from the Finnish Hospital Discharge Register (FHDR), the Cause of Death Register, and the registries of the Social Insurance Institution (SII) to include all the operations performed in Finland from 1999 to 2015 for degenerative or rheumatoid cervical disease. All of the administrative registries mentioned above utilize personal identity codes (PIC), which allows the data to be linked reliably on an individual level as well as making a differentiation between primary operations and reoperations. The method for the database construction in the PERFECT project has been elucidated in detail previously [27]. The coverage of the FHDR data has been shown to be over 95% and the FHDR based diagnosis has been confirmed by comparison with external data in 75 to 99% of the cases, with a higher likelihood of false positives for rare diseases [36]. The register of special reimbursements of the SII for the treatment of chronic illnesses was utilized to identify patients with RA and to enhance the comorbidity data provided by the FHDR. Data on the use of prescription drugs, identified by the Anatomical Therapeutic Chemical (ATC) codes, was collected from the registers of the SII.

The creation of the PERFECT Spine database was approved by the Ethics Committee of the National Institute for Health and Welfare (THL 496/6.02.00/2011), and the respective authorities of the administrative registries approved the combining of the data. As the data was acquired from the administrative registries anonymized, and the patients were not contacted, informed consent was not required. The article was constructed in adherence with the STROBE guidelines.

### Study setting and patients

**Fig. 1:**
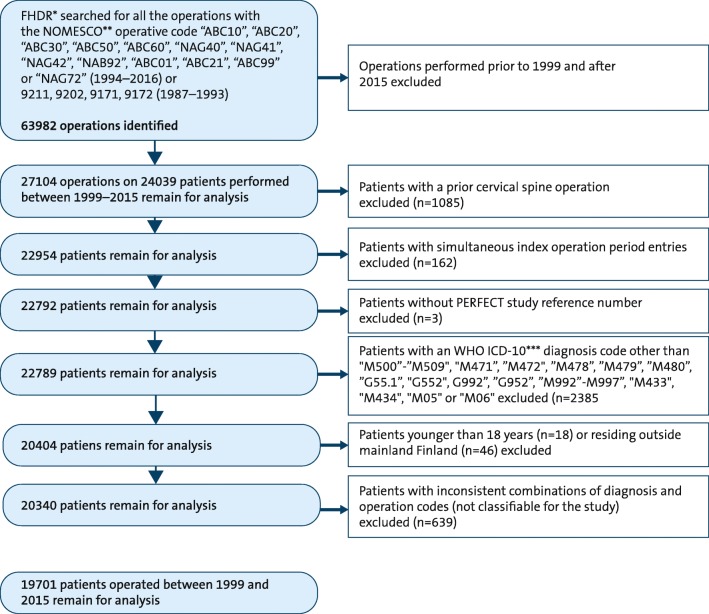
Data purification flowchart

The patients were identified from the FHDR by using the primary and secondary cervical spine operation codes from the Finnish version of the Nordic Medico-Statistical Committee Classification of Surgical Procedures (NOMESCO, http://urn.fi/URN:ISBN:978-952-245-858-2). The inclusion of each patient was further evaluated by a cross-linkage with a World Health Organization International Classification of Diseases (WHO ICD-10, the 2016 version) diagnostic code consistent with degenerative or rheumatoid cervical spine disease (http://urn.fi/URN:NBN:fi-fe201205085423). The operative and diagnostic codes used are listed in Table 1. The patients were classified into five diagnostic and three procedure groups as depicted in Table 2; the process of data purification is illustrated in Fig. 1. Each patient was entered into the database only once even if they had undergone multiple operations and were followed since the day of the first cervical operation for at least 2 years postoperatively. The comorbidity data was collected from the FHDR from 1987 until the time of the first operation for each patient individually by using the ICD-10 and the corresponding ICD-9 codes (Supplementary Table S2). From the SII registers, the comorbidities were recorded covering the year preceding the index operation by using the special reimbursement codes and the ATC-codes (Supplementary Table S3). The FHDR data and the SII data on the comorbidities were combined and comorbidity was recorded if it was documented in either of the two registers, except for epilepsy, which was diagnosed solely on the SII reimbursement code or the diagnosis code.

**Table 1:** The diagnosis (the 10th version of the World Health Organization International Classification of Diseases) and the procedure (Nordic Medico-Statistical Committee classification of surgical procedures) codes used to identify and group degenerative cervical spine patients

Diagnosis code	
Disc protrusion (intervertebral disc disorders)	
M50.0 (*G99.2)	Cervical disc disorder with myelopathy
M50.1	Cervical disc disorder with radiculopathy
M50.2	Other cervical disc displacement
M50.3	Other cervical disc degeneration
M50.8	Other cervical disc disorders
M50.9	Cervical disc disorder, unspecified
M99.5	Intervertebral disc stenosis of neural canal
M99.7	Connective tissue and disc stenosis of intervertebral foramina
G55.1*	Nerve root and plexus compressions in intervertebral disc disorders
Foraminal stenosis (bony or ligamentous obstruction)	
M47.2	Other spondylosis with radiculopathy
M99.6	Osseous and subluxation stenosis of intervertebral foramina
G55.2*	Nerve root and plexus compressions in spondylosis
Spinal canal stenosis (bony or ligamentous obstruction)	
M47.1	Other spondylosis with myelopathy
M47.8	Other spondylosis without myelopathy or radiculopathy
M47.9	Spondylosis, unspecified
M48.0	Spinal stenosis (caudal stenosis)
M99.2	Subluxation stenosis of neural canal
M99.3	Osseous stenosis of neural canal
M99.4	Connective tissue stenosis of neural canal
G95.2	Cord compression, unspecified
G99.2	Myelopathy in diseases classified elsewhere
Atlanto-axial subluxation	
M43.3	Recurrent atlanto-axial subluxation with myelopathy
M43.4	Other recurrent atlanto-axial subluxation
M05.*x*	Seropositive rheumatoid arthritis
M06.*x*	Other rheumatoid arthritis
Procedure codes for cervical operations	
Anterior decompression and fusion/prosthesis procedures	
ABC21	Anterior decompression of cervical spine with insertion of interbody fixating implant
NAG40	Anterior fusion of cervical spine without fixation
NAG41	Anterior fusion of cervical spine with fixation
NAG72	Total replacement of vertebra by reconstruction
NAB92	Replacement of intervertebral disc with prosthesis
Decompression procedures (anterior or posterior)	
ABC01	Percutaneous endoscopic discectomy for cervical intervertebral disc displacement
ABC10	Microsurgical excision of cervical intervertebral disc displacement
ABC20	Open discectomy of cervical spine
ABC30	Decompression of cervical nerve roots
ABC50	Decompression of cervical spinal canal and nerve roots
ABC60	Decompression of cervical spinal cord
ABC99	Other decompressive operation on spinal cord or nerve root
Posterior decompression and fusion procedures	
NAG42	Posterior fusion of cervical spine with or without fixation

**Table 2:** The combinations of the diagnosis (the 10th version of the World Health Organization International Classification of Diseases) and the procedure (Nordic Medico-Statistical Committee Classification of Surgical Procedures) codes used to group degenerative and rheumatoid cervical spine patients

Diagnostic group	Diagnosis codes	Procedure codes		
		Decompression only	Anterior decompression and fusion (or disc replacement)	Posterior decompression and fusion
Disc protrusion (DP)	M50.0 M50.1 M50.2 M50.3 M50.8 M50.9 G55.1	ABC01 ABC10 ABC20 ABC30 ABC50 ABC60	NAG40 NAG41 NAB92	
Foraminal stenosis (FS)	M47.2 G55.2 M99.6 M99.7	ABC30 ABC50 ABC99	NAG40 NAG41	NAG42
Spinal canal stenosis (SCS)	M47.1 M47.8 M47.9 M48.0 M99.2 M99.3 M99.4 M99.5 G95.2 G99.2	ABC30* ABC50 ABC60 ABC99	ABC21 NAG40 NAG41 NAG72	NAG42
Degenerative atlanto-axial subluxation (dAAS)	M43.3 M43.4			NAG42
Rheumatoid atlanto-axial subluxation (rAAS)	M05.*x* M06.*x* M43.3 and SII^a^ code for RA^b^ M43.4 and SII code for RA			NAG42

^a^Social Insurance Institution of Finland

^b^Rheumatoid arthritis

*A total of 688 cases with diagnosis codes consistent with spinal canal stenosis and the operative code ABC30 for foraminotomy, mostly from one hospital, were also included in the spinal canal stenosis group

Patients younger than 18 years of age, residing outside mainland Finland, or with an ICD-10 code consistent with cancer, inflammatory spondylitis other than RA, other secondary spondylarthropaties, osteoporotic fracture, congenital spinal deformity, osteochondrodysplasia, or trauma as an indication for surgery were excluded from the study. Further, patients with a previous cervical spine operation after 1986 were excluded from the data.

### Demographic data

The hierarchical system of treatment and referral in Finland is based on the area of residence, so the incidence of surgery for each hospital was calculated based on the adult population of its referral area. The number of working-age neurosurgery specialists for each year was obtained from the Finnish Medical Association.

### Statistical analyses

The population characteristics were described with proportions, means, and standard deviations. The measures of incidence were standardized for age and sex by the indirect method of standardization, calculating the observed cases against expected cases in the entire adult population of mainland Finland and comparing the observed cases to the mean of expected cases between 1999 and 2015. As data is presented for the entire population rather than a sample of the population, the use of statistical significance testing was not considered appropriate. Due to the low number of patients in the degenerative AAS group, the AAS subgroups were combined for analyses.

## Results

### Patients

Data on 24,039 patients was collected from the FHDR. After data purification, 19,701 patients were reliably identified as having undergone a primary cervical spine operation for degenerative or rheumatoid cervical spine disease in Finland between 1999 and 2015 (Fig.1). A detailed description of the patient demographics is given in Table 3. The patients that underwent operations were slightly older and more frequently male than the catchment population (Supplementary Table S4). A total of 54.6% of the operated patients were 45 to 60 years old.

**Table 3:** The baseline data of the patients operated for degenerative or rheumatoid cervical spine disease in Finland between 1999 and 2015

	Disc protrusion	Foraminal stenosis	Spinal canal stenosis	AAS^a^	All patients
Patients (*N*)	6926	6874	5580	321	19,701
Female (%)	47.9	42.4	40.7	77.3	44.4
Mean age	47.5	53.3	60.0	61.2	53.3
Age, SD	9.7	9.0	11.9	11.4	11.4
Age group (%)					
18–44	39.1	15.4	9.6	7.5	22.0
45–60	52.4	66.7	43.6	31.8	54.6
61–75	7.7	16.2	35.4	52.0	19.3
Over 75	0.7	1.7	11.3	8.7	4.2
Comorbidity (%)					
Rheumatoid arthritis	2.7	4.0	6.0	91.3	5.5
Hypertension	25.1	35.3	46.8	48.0	35.2
Atrial fibrillation	2.2	3.9	6.6	10.0	4.1
Cardiac insufficiency	0.4	0.8	2.6	6.5	1.3
Coronary artery disease	4.1	6.8	12.0	12.8	7.4
Peripheral artery disease	0.6	1.0	3.0	3.4	1.5
Hypercholesterolemia	8.8	13.5	15.0	6.5	12.1
Diabetes	6.0	7.0	12.5	6.2	8.2
Uremia	0.1	0.1	0.5	0.9	0.2
Cancer	3.2	4.2	8.0	11.5	5.1
COPD^b^ or asthma	15.3	16.8	16.9	10.3	16.2
Dementia	0.1	0.3	1.5	1.2	0.6
Demyelinating or neurodegenerative disease (other than dementia)	0.8	0.9	2.2	0.3	1.2
Parkinson’s disease	0.6	1.1	2.4	1.6	1.3
Epilepsy	1.4	1.6	2.4	2.2	1.8
Cerebrovascular disease	3.0	4.1	8.0	3.4	4.8
Depression*	19.0	22.4	20.0	14.3	20.4
Other mental disorder	3.1	3.7	5.0	1.2	3.8
Alcohol/drug addiction	3.4	4.5	4.7	2.2	4.1
Arthrosis of the hip or knee joint	1.1	2.6	6.1	3.1	3.1
Arthrosis of the shoulder joint	0.2	0.5	0.2	0	0.3
Rotator cuff syndrome	6.1	10.4	6.3	2.2	7.6
Fibromyalgia	0.1	0.1	0.1	0	0.1
Hospital status (%)					
Public	93.6	97.9	93.4	84.7	94.9
Private	6.4	2.1	6.6	15.3	5.1

^a^*AAS* atlanto-axial subluxation

^b^*COPD* chronic obstructive pulmonary disease

*Prevalence of depression is likely vastly overestimated. Comorbidity recording is based on the use of antidepressants, which in this patient group may be used for neuropathic pain

### Operation incidences and indications

The mean operation incidence over the study period was 27.6/100,000 people. The indication for operation was disc protrusion (DP) in 35.2% of the operations, foraminal stenosis (FS) in 34.9%, and spinal canal stenosis (SCS) in 28.3% of the operations. The overall operation incidences in each diagnostic group are given in Table 4. The overall age- and sex-adjusted rate of surgery for radicular symptoms (DP and FS groups, excluding the patients with the ICD-10 diagnosis code M50.0) was 19.3/100,000 people and for degenerative cervical myelopathy (DCM) (SCS-group, excluding the patients with the diagnosis codes M47.8 and M47.9) 6.6/100,000 people (Table 4). A total of 94.9% of the operations were performed in public hospitals.

**Table 4:** The annual mean age and gender distribution of the patients, the age- and sex-adjusted incidences of surgery (operations/100,000 people aged 18 years or older) overall and in each diagnostic group, the incidences of surgery for radiculopathy or myelopathy, the incidences of surgery in each age group and each hospital and the frequency of neurosurgery specialists in Finland

	1999	2000	2001	2002	2003	2004	2005	2006	2007	2008	2009	2010	2011	2012	2013	2014	2015	Overall
Operation count (*n*)	832	945	779	899	1002	900	1117	1028	1124	1245	1251	1281	1453	1466	1576	1433	1370	19,701
Mean age of the patients (*y*)	52.5	51.2	52.0	52.1	52.1	52.7	52.9	53.8	53.7	52.8	53.5	53.3	54.1	53.7	53.8	54.8	54.3	53.3
SD	11.9	11.4	11.8	10.4	11.0	11.0	10.8	11.4	11.1	11.1	11.0	11.1	11.3	11.4	11.7	11.9	11.8	11.4
Female (%)	43.3	43.2	43.5	39.2	41.0	43.7	44.2	43.7	44.1	44.9	46.8	46.1	44.8	46.9	44.7	46.4	44.3	44.4
Overall incidence of surgery	21.0	23.6	19.2	22.0	24.3	21.7	26.7	24.5	26.6	29.4	29.4	30.0	33.9	34.1	36.5	33.1	31.7	27.6
Disc protrusion	7.8	10.7	9.0	9.1	9.0	8.3	9.4	7.9	8.2	10.1	10.4	9.7	11.5	11.5	11.1	10.7	10.8	9.7
Foraminal stenosis	5.3	5.3	4.1	6.6	7.9	6.4	9.0	8.0	9.7	11.1	11.0	11.9	12.8	12.5	15.4	13.5	12.4	9.6
Spinal canal stenosis	6.9	6.6	5.2	5.6	6.5	6.6	7.8	8.3	8.6	7.9	7.8	8.0	9.4	9.9	9.9	8.7	8.4	7.8
Degenerative AAS (*n*)	0.1 (4)	0.1 (3)	0.1 (3)	0.1 (2)	0.1 (1)	0.1 (3)	0 (2)	. (0)	. (0)	0 (1)	. (0)	0 (1)	0 (1)	0 (1)	0 (1)	0.1 (3)	0 (1)	0
Rheumatoid AAS (*n*)	1.0 (37)	0.8 (32)	0.9 (34)	0.6 (23)	0.9 (33)	0.4 (16)	0.5 (20)	0.3 (13)	0.2 (7)	0.4 (15)	0.2 (9)	0.4 (16)	0.2 (9)	0.1 (5)	0.1 (5)	0.2 (11)	0.1 (3)	0.4
Incidence of surgery for radiculopathy	13.1	16.1	12.4	15.7	16.9	14.5	18.3	15.8	17.8	21.1	21.4	21.6	24.3	23.9	26.5	24.1	23.3	19.3
Incidence of surgery for myelopathy	5.8	5.5	5.1	4.5	5.4	6.0	7.1	7.5	7.2	6.5	6.7	6.4	7.3	8.3	8.3	6.9	7.0	6.6
Incidence of surgery in each age group																		
18–44	11.5	14.5	11.0	11.2	13.4	11.4	13.4	11.7	12.0	15.6	14.1	15.5	15.5	16.3	18.8	15.3	16.3	14.0
45–60	36.5	42.0	33.4	43.6	5.1	40.9	51.4	44.8	52.2	57.2	58.5	57.4	66.3	68.4	69.8	62.6	59.8	52.3
61–75	23.5	22.2	20.9	20.1	23.9	20.8	27.1	28.7	27.4	27.2	27.7	30.4	34.6	30.9	35.5	35.5	32.0	28.2
Over 75	11.6	9.3	9.7	7.5	10.1	11.5	10.0	12.4	13.2	11.3	13.7	11.7	17.4	17.6	17.9	18.9	15.2	13.2
Incidence of surgery in each hospital																		
Helsinki	20.5	26.7	20.9	23.5	28.9	20.7	28.6	25.2	27.5	29.2	26.3	22.8	27.4	26.6	31.3	21.3	29.5	25.8
Kuopio	29.4	32.8	27.1	30.8	30.8	31.6	38.7	41.0	47.8	47.8	43.0	45.5	61.9	55.6	62.7	59.6	49.3	43.1
Oulu	17.4	18.3	12.0	22.3	16.7	19.9	19.8	17.9	19.8	21.5	22.9	26.9	30.6	26.9	28.5	31.7	21.6	22.1
Tampere	20.5	22.5	17.2	19.1	20.8	21.4	25.9	21.4	20.5	29.9	35.9	34.1	34.4	39.4	44.2	42.3	35.8	28.5
Turku	16.3	12.1	16.2	12.4	18.9	14.6	15.6	14.0	15.9	14.6	16.4	26.3	20.5	26.1	19.8	23.9	2.9	18.3
Neurosurgeon frequency*	1.1	1.2	1.2	1.3	1.3	1.4	1.4	1.4	1.5	1.7	1.7	1.8	1.8	1.9	1.9	1.9	2.1	

*Number of neurosurgery specialists/100,000 people aged 18 years or older

### Trends over time

**Fig. 2:**
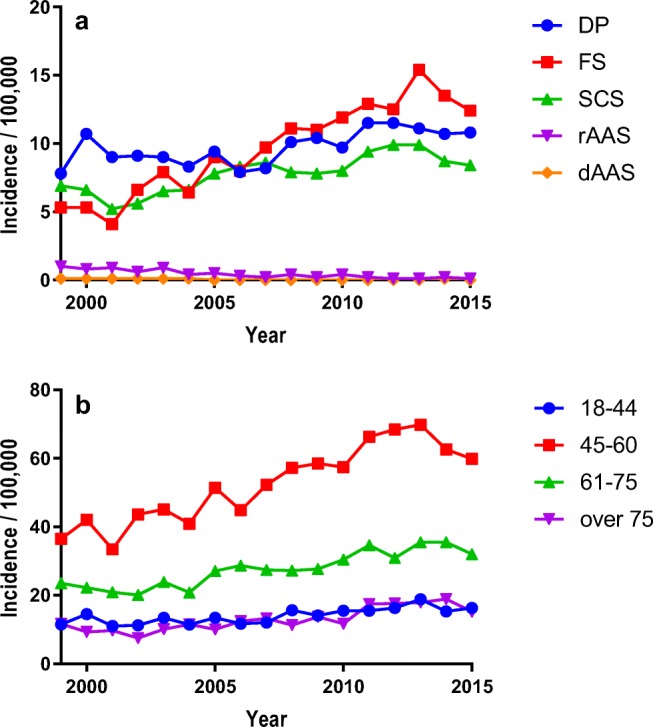
The adjusted overall incidence of operations in each diagnostic (**a**) and age (**b**) group (observe the differences in the scaling between **a** and **b**). DP, disc protrusion; FS, foraminal stenosis; SCS, spinal canal stenosis; rAAS rheumatoid atlanto-axial subluxation; dAAS, degenerative atlanto-axial subluxation

**Fig. 3:**
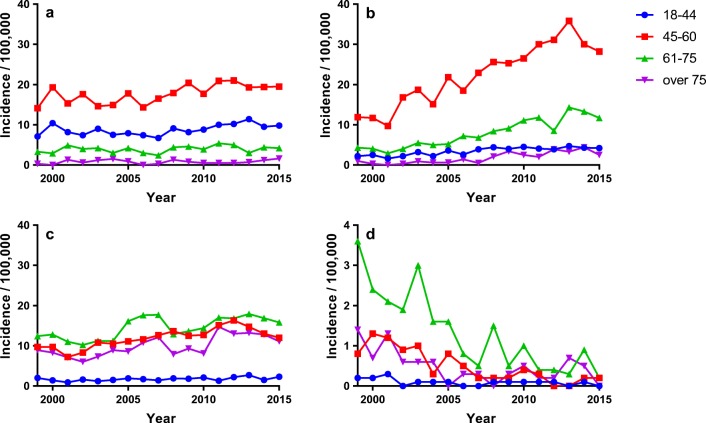
The adjusted incidences of operation for disc protrusion (**a**), foraminal stenosis (**b**), spinal canal stenosis (**c**), and rheumatoid atlanto-axial subluxation (**d**) in each age group (observe the differences in the scaling between **a**–**c** and **d**)

The adjusted incidence of operations varied between 19.2 and 36.5 per 100,000 people. An increasing trend from 1999 to 2013 was observed, but the incidence plateaued thereafter. The increase in the operation rates was the highest for FS (134%, Table 4, Fig. 2a). For DP, the rise in the incidence of surgery was slight compared with SCS and especially FS (Table 4, Fig. 2a). Of the patient groups, only operations for AAS decreased, with operations for rAAS declining from 37 per annum in 1999 to a mere 3 in 2015. The incidence of surgery for degenerative AAS remained very low. The adjusted incidence of operations for radiculopathy rose from 13.1 to 23.3/100,000 people. The annual incidences of operations for myelopathy varied between 5.8 and 8.3/100,000 people; however, a slightly increasing trend was observed. The operation incidences are given in Table 4 and illustrated in Fig. 2a and b. The most marked increase was in the 45-to-60-year age group and especially for FS (Table 4, Fig. 3a–d). The mean age of the patients rose from 52.5 ± 11.9 to 54.3 ± 11.8 years, while the mean age of the catchment population rose from 47.2 to 49.9 years (Supplementary Table S3). The percentage of operations performed in private hospitals remained low, between 1.8 and 5.6%, with the exception of the years 2004–2007, when 11.3 to 14.5% of the operations were performed in private hospitals. The number of working neurosurgery specialists rose from 45 in 1999 to 92 in 2015 (1.1/100,000 to 2.1/100,000 people aged 18 years or older).

### Regional differences

**Fig. 4:**
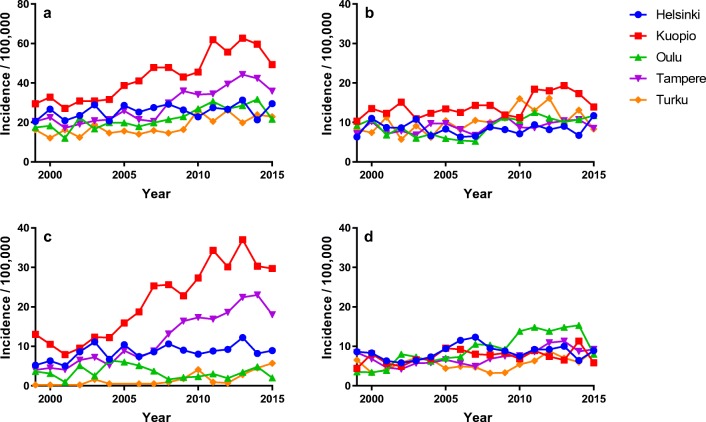
The adjusted incidences of operation in each university hospital overall (**a**), for disc protrusion (**b**), for foraminal stenosis (**c**), and for spinal canal stenosis (**d**) (observe the differences in the scaling between **a** and **b**–**d**)

There was over a 2.5-fold difference in the overall adjusted operation incidence between the highest and the lowest rate university hospitals (43.1 and 18.3 operations/100,000 people; Table 4, Fig. 4a). The differences in the incidences were most pronounced in the FS group with the overall incidences ranging from 1.6 to 21.1 per 100,000 people (Fig. 4b). The incidence of surgery for FS rose in all five university hospitals, but the differences remained substantial (Table 4, Fig. 4b). The regional incidences in each diagnosis group are illustrated in Fig. 4a–d. There were also differences between the tertiary level hospital regions within some of the university hospital catchment areas (Supplementary Fig. S5).

## Discussion

### Key results

The age- and sex-adjusted incidence of surgery for cervical degeneration or rheumatoid cervical disease rose from 21.0 to 36.5/100,000 people between 1999 and 2013 and plateaued thereafter. The rise was the most pronounced in the foraminal stenosis group and in the 45-to-60-year age group (Fig. 3a–d). The incidence of surgery for radiculopathy increased by 77.9%, from 13.1 to 23.3 operations/100,000 people, while the operations rates for DCM increased by only 20.7%, from 5.8 to 7.0 operations/100,000 people. Of the five diagnostic groups, surgery for FS became the most common indication by 2006, with a mean incidence of 13.3/100,000 at 2011–2015. During the same period, the mean incidences of surgery for DP and SCS were 11.1/100,000 and 9.3/100,000 patients. The overall operation incidences varied from 18.3 to 43.1 operations/100,000 people between the highest and the lowest incidence university hospital catchment area; the variance being the highest for FS. The operations for rheumatoid AAS very nearly ceased during the study period.

### Indications for surgery

Approximately 35% of the operations were performed for disc protrusion, 35% for foraminal stenosis, and 28% for spinal canal stenosis. Over the 17-year period, the operations for FS especially increased, while the incidence of operations for DP only slightly increased. The distribution in the diagnoses is similar to the findings from Norway, where 79% of the operations were performed for radiculopathy and 21% for myelopathy [14]. However, in a recent study of all the cervical fusions in the state of New York, 70% of the operations were found to be performed for degenerative disc, 30% for spondylosis, and only 13% for spinal stenosis [32]. This may suggest differences in the operation indications between Finland and the US, particularly in the operations for axial neck pain, for which the indications are controversial [8, 30]. In our analysis, the operation indication was coded as axial neck pain (ICD-10 codes M47.8, M50.2 - M50.9) in only 299 operations, representing 1.5% of all the operations.

### Trends in the incidence of surgery

The rate of surgery in Finland appears to be slightly higher than the rate found in Norway but is only 25 to 48% of the reported estimated incidence of surgery in the US which, however, are not restricted to primary operations [14, 18, 22, 24, 26]. The differences in the data sources and the inclusion criteria make direct comparisons of the rates somewhat uncertain. Finland and Norway have similar tax-funded hierarchical health care systems (The Health Systems and Policy Monitor, www.hspm.org). In both Finland and Norway, the primary care physicians are the gatekeepers for a specialist consultation. In Finland, MRI scanning is reimbursed by the SII only if there is a referral from a specialist. These factors limit the access to imaging and specialist consultation perhaps more strictly than in insurance-funded systems. Insurance status has previously been proven to influence the operation rates for DCSD [1, 2]. The prevalence of the DCSD symptoms or the patient attitudes toward surgery may be different between these two North-European countries and the US. The prevalence of the symptoms of neural compression has not been studied in Finland. However, in a nationally representative cluster sample of Finnish people aged 18 years or older, 40% of women and 26% of men over the age of 30 years reported having experienced neck pain during the past month [17], which is consistent with the 15.4 to 45.3% prevalence reported internationally [11]. The operation indications may also be different, as indicated by the distribution of operative diagnoses found in New York [32] compared with Finland or Norway [14]. It has also been suggested that the increase in the operation rates and the inclusion of fusion in the US might be due to the surgeons’ financial interests [2, 24]. In Finland, the surgeons working in the public hospitals receive a fixed salary independent of the number or type of operations they perform.

The age- and sex-adjusted operation rates increased by 76% in Finland between 1999 and 2013 and plateaued thereafter. The rate of surgical hospitalizations in general has remained stable between 1995 and 2010 [21]. In the US, the rate of surgery for DCSD appears to have more than tripled between 1990 and 2013 and seems to have plateaued at 72 to 75 operations per 100,000 people [18, 22, 24, 26]. The increase in Finland was the most pronounced in the FS group, while the increase in surgery for SCS or DP was lower. The operations increased especially in the middle-age, working-age patient group, which is in concordance with the increase in surgery for FS and the progression of degeneration with age [25]. In Norway, the operation incidence increased especially in the oldest age groups [14]. In the US, the mean age of the patients has risen [1, 22, 24, 26] while in Finland, the mean age of the patients actually rose less than in the catchment population. The rise in surgery for radiculopathy (a 77.9% increase in the adjusted operation frequency between 1999 and 2015) and particularly for FS, compared with a 20.7% increase in operation rates for DCM, illustrates a shift in the operative indications. In Norway, operations for radiculopathy increased by 86.5% and for DCM by 74.1% between 2008 and 2014 [14]. In the US, operations for CSM increased from 0.6 to 4.1/100,000 people between 1993 and 1997 [16]. In Finland, the number of neurosurgery specialists almost doubled during the study period, which may have influenced the availability of neurosurgical evaluation and treatment.

Operations for rAAS declined by 90%. The incidence of seropositive RA did not change and only the incidence of seronegative RA in females declined in Finland between 2000 and 2007 [28]. The introduction of disease-modifying anti-rheumatic medications has induced a decline in the rates of joint arthroplasty [20, 23, 35] and most likely influenced the decline in rAAS surgery. In the US, the rates of surgery for rAAS have also declined [35].

The operative techniques changed as well during the study period. In 1999, most of the operations were decompressions without fusion. By 2006, anterior cervical decompression and fusion (ACDF) became the most commonly used technique and in 2015, and 85% of the operations were ACDF. A detailed discussion of the changes in the operative techniques is provided separately (reference to a separately submitted manuscript).

### Regional differences

The rates of surgery varied by over 2.5-fold between the five university hospitals (Table 4), but much less within the catchment areas (Supplementary Fig. S5). The differences were particularly marked in the FS group. The myelopathic symptoms of SCS or motor weakness, which is more common in DP, may more consistently be considered an indication for operative treatment than the more prevalently sensory symptoms of spondylotic FS [34]. There may also be regional differences in the prevalence of symptomatic DCSD. Similar regional differences in cervical spine surgery have been found in the US and Norway both at a county level and between the different parts of the countries as well [1, 2, 9, 14, 18, 37]. In a population-based study of lumbar spinal surgery, the factors influencing the rate of surgery were the surgeons’ keenness and the presence of MRI scanners, while the supply of surgeons or family physicians, the prevalence of the disease, or the patients’ enthusiasm was not significantly related to surgical rates [4]. In Finland, regional differences have been detected for instance in the rate of hip and knee arthroplasty as well as lumbar discectomy [13, 19]. Regional differences in the rates of various elective operations have been reported internationally as well and seem to remain relatively stable over the decades [5]. The differences in the regional operation rates probably reflect the lack of evidence on the superiority of different treatment modalities in the varying clinical scenarios of cervical degenerative disease. Prospective clinical series demonstrate the effectiveness of surgical treatment whereas the indications are often elusive in the continuum of degenerative changes and leave room for clinical judgment and local practice policies. Based on our data, it is not possible to ascertain the prevalence of radiculopathy or myelopathy in the population, nor the optimal rate of surgery.

### Strengths and limitations of the study

Every patient, from all the hospitals in Finland, undergoing an operation and fulfilling the inclusion and the grouping criteria was included in the study. The selection bias inherent to retrospective studies was probably avoided as the exclusion of the patients with incomplete data occurred presumably in a random manner. The Finnish public health care system is available to all residents and is tax-funded with a negligible cost to the patients, thus permitting access to operative treatment regardless of the financial circumstances of the patients. The private hospitals are independent of the area of residence and covered by occupational health plans, private insurance, and partly by the SII, but only 5.5% of the operations occurred in the private hospitals. Therefore, we assume that the risk of referral bias in the analysis of the regional rates is low. The personal identity codes make the data compiling accurate and facilitate the ability to distinguish between the primary operations and the reoperations. Further, the precise administrative registries enable reliable operation rate adjustments for changes in the age and gender distribution over the study period.

Most of the weaknesses are those inherent in studies utilizing administrative databases. Most importantly, pertinent clinical information, including the severity or the duration of the symptoms, cannot be determined from the administrative data. Consequently, the actual operative indications cannot be acquired and we can only speculate on the changes in time or the differences between regions. Furthermore, data on possible confounders, such as socioeconomic, educational, or smoking backgrounds of the patients is lacking.

A combination of the procedure and the diagnostic codes for the inclusion of the patients in the database was used to amend the possible non-systematic coding errors, and patients were excluded if the operative and the diagnostic codes could not be matched (603 operations). However, 688 patients had diagnostic codes classified as SCS and an operative code for foraminotomy (ABC30). These were mostly from one hospital at the beginning of the study period and exceeded the number of patients with the diagnosis code for FS (M47.2). These cases were classified as SCS. Further, the code for lumbar spinal stenosis (M48.0) has been traditionally used in some hospitals for cervical SCS (1156 operated patients with the IDC-10 code M48.0 were identified) and was included in the diagnostic criteria. Other systematic differences in the use of the ICD-10 codes between the university hospitals may also exist. Differences may be suspected especially in the coding for FS and SCS, which may have caused the national incidence of surgery for FS to be lower and for SCS higher than the actual incidence. The coding practices seem to have remained unchanged; the trends are likely to be more reliable than the distribution of the diagnoses.

### Generalizability

Every primary operation performed in Finland over the study period was included, with presumably low selection bias. The prevalence of DCSD may be different between different populations, as differences in the incidence of radiculopathy [33] and the operative rates between different ethnic groups [1, 10] have been found. These results represent trends that are independent of change in the population age or sex distribution as well as the surgeon’s income or the insurance coverage.

### Conclusions

The age- and sex-adjusted rate of surgery for degenerative diseases of the cervical spine has increased from 20.7 to 36.5 operations per 100,000 people aged 18 or older in Finland between 1999 and 2013 and plateaued thereafter at slightly over 30 operations/100,000 people annually. The incidence is approximately half of the estimated incidences found in the US, which may be assumed to be explained somewhat by the differences in the frequency of operations for axial neck pain. During the last 5 years, the incidence of surgery for radiculopathy reached the mean of 24.4 operations/100,000 people, while the mean operation incidence for DCM was 7.6 operations/100,000 people. Of the specific diagnostic groups, the age- and sex-adjusted rate of surgery for both cervical disc protrusion and spinal canal stenosis increased moderately, both of them fairly clearly defined conditions based on the symptoms and the radiological findings. However, the rate of surgery for foraminal stenosis, a more chronic and ambiguous entity with less clear benefit from surgery, increased by nearly 140%, from 5.2 to 12.4 operations per 100,000 people, and exceeded the rate of surgery for both DP and SCS by over twofold. This is likely due to a change in the operative indications and perhaps the patient expectations. Surgery for rheumatoid AAS has almost disappeared, likely due to the effect of the immunomodulatory drugs. There are vast regional differences in the rates of surgery in Finland, reflecting the lack of convincing scientific evidence and consequently of clear guidelines for surgical indications and techniques.

## Electronic supplementary material

ESM 1 (PDF 268 kb)

## Abbreviations

AAS: atlanto-axial subluxation
ACDF: anterior cervical decompression and fusion
ATC: Anatomical Therapeutic Chemical
CSM: cervical spondylotic myelopathy
DCM: degenerative cervical myelopathy
DCSD: degenerative cervical spine disease
DP: disc protrusion
FHDR: Finnish Hospital Discharge Register
FS: foraminal stenosis
ICD: International Classification of Diseases
NOMESCO: Nordic Medico-Statistical Committee Classification of Surgical Procedures
PIC: personal identity code
RA: rheumatoid arthritis
rAAS: rheumatoid atlanto-axial subluxation
SCS: spinal canal stenosis
SII: Social Insurance Institution of Finland
US: United States
WHO: World Health Organization
