# Supplementary material for: Surgery for degenerative cervical spine disease in Finland, 1999–2015
Source: Acta Neurochir (Wien). 2019 Jun 1;161(10):2147–59. doi: 10.1007/s00701-019-03958-6 (PMC6739276; doi:10.1007/s00701-019-03958-6)
Supplement: Supplementary file 1 — (PDF 268 kb) [file 701_2019_3958_MOESM1_ESM.pdf]

Electronic supplementary material to:
Surgery for degenerative cervical spine disease in Finland, 1999 – 2015.

Supplementary Table S1. Review of the previous literature on the rates of cervical spine surgery.

	Author(s)	Study years	Data source and study population	Codes used to identify patients	Rate of surgery	Changes in the operative techniques	Demographic trends
**Degenerative cervical spine only**	Einstadter, D., et al (1993) [5]	1986 - 1989	CHARS[a]; all **discharges** of residents of Washington state ≥ 20 years of age, excluding cases of neoplasm, spinal infection, trauma, inflammatory spondylarthropathy, congenital anomalies and cases unrelated to cervical spine	Diagnosis (ICD-9-CM[b]): 721.0, 721.1,721.90, 721.91, 722.0, 722.70, 722.71, 722.2, 722.4, 722.6, 722.8, 722.81, 722.90, 722.91, 723.0, 723.1, 723.2, 723.4, 723.5, 723.7, 723.8, 723.9, 738.2, 738.4, 739.1, 837.0, 847.9, Procedure: 03.0, 03.02, 03.09, 03.6, 80.50, 80.51, 80.59, 81.00, 81.01, 81.02, 81.08, 81.09	Crude rate: 33/100,000 – 39/100,000 Discectomy ± fusion 23.2/100,000 Cervical disc herniation 22/100,000	Distribution of diagnoses and procedures remained constant: discectomy and fusion 43 %, discectomy 23 %, fusion w/o discectomy 16.7 %, laminectomy 13.8 %	Not analyzed
	Patil, P., et al. (2005) [12]	1990 - 2000	NIS[c]; all **hospitalizations** of patients ≥ 18 years of age with cervical-specific diagnostic and surgical procedure codes; trauma-related, infection-related, and neoplastic diagnoses excluded	Diagnosis(ICD-9-CM): 344.01-04, 344.2, 344.4, 353.0, 353.2, 721.0, 721.1, 722.0, 722.4, 722.71, 722.81, 722.91, 723, 723.0-4, 723.7-9, 738.2, 739.1, 741.01, 741.91, 756.16, 805.0, 805.1, 806.0, 806.1, 839.0, 839.1, 847.0, 952.0, 953.0, 953.4 Procedure: 03.0, 03.02, 03.09, 03.6, 78.69, 80.5, 80.50, 80.51, 80.59, 81.0, 81.00-81.09	29/100,000 – 55/100,000 (normalized to US census data); Males: 33.5/100,000 – 58.5/100,000 Females: 25.0/100,000 – 58.5/100,000	ACDF[d] 17.8 – 69.5 % PDF[e] 0.3 – 3.8 % PD[f] 70.5 – 24.6 %	Mean age: 47.5 – 49.2 Female: 44.8 – 48.5 % CCI[g]: patients with no comorbidity: 91.3 – 85.5 %; CCI ≥ 3: 4.0 – 5.3 %
	Oglesby, M., et al. (2013) [11]	2002 - 2009	NIS; all **hospitalizations** of patients ≥ 18 years of age with anterior or posterior cervical fusion or decompression for degenerative etiologies	Diagnosis(ICD-9-CM): 721.0-1, 722.0, 722(IC)-9-CM).4, 722.71, 722.81, 722.91, 723.x Procedure: 81.02, 81.03, 03.09	52.2/100,000 – 60.8/100,000 (p=0.047; population-adjusted)	ACDF 80.9 – 81.2 % (p=0.021) PDF 6.5 - 9.5 % (p<0.0005) PD 12.6 - 9.3 % (p=0.01)	Mean age: 50.7 – 54.1 (p<0.0005) Female: 48.8 – 49.6 % (n.s.) CCI: 1.97 – 2.44 (p<0.0005)
	Marquez-Lara, A., et al. (2014) [10]	2002 - 2011	As above	As above	Significant increase in total count of operations (p<0.001); rates not given	ACDF 85.4 – 82.5 % PDF 4.1 – 9.1 % (p<0.001) PD 10.4 – 8. 4 %	Mean age: 50.7 – 55.6 (p<0.001) Female: 48.8 – 50.8 % (n.s.) CCI: 1.97 – 2.61 (p<0001)
	Kristiansen, J-A., et al. (2016) [6]	2008 - 2014	Norwegian Patient Registry, the registry of Oslofjord Clinic private hospital and the telephone survey information of other relevant private clinics; all admissions for surgery for degenerative	Diagnosis (ICD-10[h]): M47.1, M47.2, M47.21, M47.22, M47.23, M47.8, M47.81, M47.82, M47.83, M48.0, M48.01-03, M48.02, M50.0, M50.1, M50.8, M50.9, M53.0, M53.1, M54.2, M99.3-7, G82.1, G82.4,	16.9/100,000 – 29.4/100,000 rates of operations for radiculopathy: 12.1/100,000 – 22.6/100,000 rates of operations for myelopathy: 4.7/100,000 – 6.8/100,000	Not analyzed	Rate for patients aged 50-55: 36.8/100,000 – 70.1/100,000 Rate for patients aged 75-80: 7.4/100,000-23.7/100,000

			changes or myelopathy excluding patients with diagnoses of neoplasms, trauma or primary infections	G83.0, G83.2, G95.2, G99.2<br>Procedure (NOMESCO[i]): ABC01, ABC10, ABC20, ABC21, ABC30, ABC50, ABC60, ABC99, NAB90, NAB91, NAC90, NAG00, HAG10, NAG30, NAG40, NAG60, NAG70, NAK10, NAR90, NAF90, NAG20			
	Liu, C., et al (2017) [8]	2001-2013	NIS; all patients ≥ 18 years of age identified by cervical spine procedure codes for degenerative disease	Diagnosis: (ICD-9-CM): 721.0-1, 722.0, 722.4, 722.71, 722.81, 722.91, 723.x<br>Procedure: 81.02, 81.03, 03.09 in absence of fusion codes 81.02 or 81.03, 81.02 and 81.03 for combined anterior-posterior fusion	75.34/100000 – 72.20/100000	ACDF: 62.39/100,000 – 56.21/100,000;<br>PDF: 2.86/100,000 – 7.81/100,000<br>Anterior-posterior cervical fusion: 0.43/100,000 – 1.86/100,000;<br>PD: 9.66/100,000 – 6.31/100,000	
Elderly	Wang, M., et al. (2009) [15]	1992 - 2005	Medicare Claims and Enrollment data Part A; beneficiaries **≥ 65 years** of age with a definite degenerative cervical diagnosis and a cervical spine procedure code	Diagnosis (ICD-9-CM): 722.0, 721.0, 721.1, 722.4, 722.71, 723.0, 723.1, 723.4, 723.7, 738.2<br>Procedure: 03.09, 80.50, 80.51, 80.59, 81.00, 81.09, 77.70, 77.77, 77.79, 78.00, 78.07, 78.09	Not analyzed	Fusion: 14.7/100 000 – 45/100,000 beneficiaries ($p<0.05$)<br>ACDF 12.6/100,000 – 35.8/100,000<br>PDF 1.1/100,000 – 6.2/100,000<br>PD 8.8/100,000 – 9.3/100,000<br>Discectomy 3.4/100,000 – 1.2/100,000	Not analyzed
Anterior surgery only	Angevine, P., et al. (2003) [2]	1990 - 1999	NHDS[j]; all **hospitalizations** of patients ≥ 15 years of age operated anteriorly for degenerative cervical disc disease	Diagnosis (ICD-9-CM): 721.0, 721.1, 722.0, 722.4, 722.71, 722.91, 723.0, 723.3<br>Procedure:80.50, 80.51, 81.00, 81.02, 77.70, 77.79	No significant change in the rate of surgery (adjusted for age)<br><br>Moderate increase (1.7) in the odds of surgery for hospitalized patients	ACDF increased significantly: 40 % for males and 62 % for females<br><br>The percentage of surgery performed with fusion increased significantly for both genders<br><br>Odds of inclusion of fusion 4.1 higher in 1999 compared to 1990	Not analyzed
	Alosh, H., et al. (2009) [1]	1992 - 2005	NIS; all **hospitalizations** of patients ≥ 18 years of age with anterior cervical spine surgery diagnosis and procedure codes	Diagnosis (ICD-9-CM): 721.0, 721.1, 722.0, 722.4, 722.71, 722.91, 723.0, 723.3<br>Procedure: 80.50, 80.51 and/and not 81.00, 81.02	(standardized to US census data):<br>males 13.1/100,000 (1992-1995) – 23.6/100,000 (2001-2005)<br>females:10.5/100,000 (1992-1995) – 23.5/100,000 (2002-2005)<br>more than 235 % increase maintaining all covariates constant in a Poisson regression model	Not analyzed	Mean age: 48.1 (1992-1995) – 51.0 (2001-2005)<br>Female: 47.9 % (1992-1995) - 51.7 % (2001-2005)<br>CCI: 0.24 (1992-1995) – 0.39 (2001-2005)

Fusion surgery for cervical spondylotic myelopathy (CSM)	Lad S., et al. (2009) [7]	1993 - 2002	NIS; all hospitalizations of patients with the diagnosis code of CSM	Diagnosis (ICD-9-CM): 721.1<br>Procedure: Clinical classifications Software code 158	Admissions for CSM: 3.73/100,000 – 7.88/100,000<br>Fusions for CSM: 0.6/100,000 – 4.1/100,000 (normalized to US population)	Fusions for CSM: 0.6/100,000 – 4.1/100,000 (normalized to US population)	Age distribution: no significant change between age groups<br>Gender distribution: no significant change<br>Comorbidity: number of patients with comorbidities and number of comorbidities per patient increased significantly
**Cervical spine surgery for any indication**	Davis, H. (1994) [4]	1979 - 1990	NHDS[j]; all **hospitalizations** of patients ≥ 25 years of age, with surgical procedure codes for spinal fusion, excision or destruction of an intervertebral disc, or exploration and decompression of spinal canal structures	Diagnosis (ICD-9-CM):<br>81.0, 81.00-81.09; 80.5. 80.50-80.59<br>Procedure:<br>03.0, 03.01-03.09	For cervical spine (adjusted for age):<br>Males: 39/100,000 – 58/100,000<br>Females: 24/100,000 – 40/100,000	Fusion: males 21/100,000 – 36/100,000; females 12/100,000-25/100,000<br>Disc: Males 11/100,000 – 14/100,000; females 7/100,000-11/100,000<br>Exploration/ decompression: Males 7/100,000 – 8/100,000; females 5/100,000 – 4/100,000	Not analyzed
	Zeidman, J. (1997) [16]	1989 - 1993	CSRS[k] databank data (data from 30-35 practicing clinicians/year; 700 – 1451 patients/year): cervical spondylosis, disc herniation, trauma, OPLL, tumor, rheumatoid arthritis, ankylosing spondylitis	Not reported	Not analyzed	Incidence of ACDF increased, discectomy only decreased<br>Posterior decompression sdecreased, PDF increased<br>Use of plates increased from 1 to 12 % of operations	Not analyzed
Cervical spine fusion for any indication	Cowan J., et al (2006) [3]	1993 - 2001	NIS; all hospitalizations of patients with surgical procedure codes for spinal fusion; only cervical data reviewed here	Diagnosis (ICD-9-CM): 721.0-722.9, 723.0, 724.0<br>Procedure: 81.01-81.03	Fusion: 7/100,000 – 38/100,000	Fusion: 7/100,000 – 38/100,000	Not reported
	Salzmann S., et al (2018) [13]	1997 - 2012	SPARCS; all primary isolated cervical fusions in patients ≥ 18 years of age; patients with atlanto-axial, subcervical or unspecified level fusions excluded	Procedures (ICD-9-CM): 81.02, 81.03	Fusion: 23.7 – 50.6 /100,000<br>Anterior fusion: 21.1 – 42.2/100,000<br>Posterior fusion: 2.21 – 6.81/100,000<br>Circumferential fusion: 0.39 – 1.4/100,000;<br>The rate of surgery increased especially for degenerative disc, spondylosis and spinal stenosis indications	Fusion: 23.7 – 50.6 /100,000<br>Anterior fusion: 21.1 – 42.2/100,000<br>Posterior fusion: 2.21 – 6.81/100,000<br>Circumferential fusion: 0.39 – 1.4/100,000;<br>The rate of surgery increased especially for degenerative disc, spondylosis and spinal stenosis indications	No change in overall fusion rate or posterior fusion rate in patients aged 18-34 years, increased for patients aged ≥ 35 years and especially in the >60-year-old patients;<br>Circumferential fusion rate increased especially in the >50-year-old patients

Anterior or posterior cervical decompression and fusion for any indication	Marawar, S., et al (2010) [9]	1990 - 2004	NHDS[j]; all ACDF procedures for any indication (patient age 2-93)	Procedure codes 81.02 (ACDF) 81.03 (PDF)	Changes between 1990 – 1994 and 2000 – 2004 ACDF 23/100,000 – 157/100,000 PDF 5/100,000 – 18/100,000	Changes between 1990 – 1994 and 2000 – 2004 ACDF 23/100,000 – 157/100,000 PDF 5/100,000 – 18/100,000	Changes between 1990 – 1994 and 2000 – 2004 Males: 57.65 % -52.46 % Average age: 47.2 – 50.48 y
**Cervical spine surgery for rheumatoid cervical disease**	Stein, B., et al (2014) [14]	1992 - 2008	NIS; all hospitalizations for operations with operation codes for spinal fusion	Diagnosis code (ICD-9-CM) 714.0 Procedure codes 81.01-81.03	C1-C2 fusion: 0.13/100,000 – 0.09/100,000 (1992-2008) ACDF: 0.06/100,000 – 0.51/100,000 Posterior fusion: 0.105/100,000 – 0.148/100,000	C1-C2 fusion: 0.13/100,000 – 0.09/100,000 (1992-2008) ACDF: 0.06/100,000 – 0.51/100000 Posterior fusion: 0.105/100,000 – 0.148/100,000	

[a]Comprehensive Hospital Abstract Reporting System
[b]International Classification of Diseases - 9th revision - clinical modification
[c]Nationwide Inpatient Sample
[d]Anterior cervical decompression and fusion
[e]Posterior decompression and fusion
[f]Posterior decompression without fusion
[g]Charlson Comorbidity Index
[h]International Classification of Diseases – 10th revision
[i]Nordic Medico-Statistical Committee
[j]National Hospital Discharge Survey
[k]Cervical Spine Research Society
[l]The New York Department of Health Statewide Planning and Research Cooperative System

References for the literature review

(1)Alosh H, Riley LH,3rd, Skolasky RL (2009) Insurance status, geography, race, and ethnicity as predictors of anterior cervical spine surgery rates and in-hospital mortality: an examination of United States trends from 1992 to 2005. Spine (Phila Pa 1976) 34:1956-1962

(2)Angevine PD, Arons RR, McCormick PC (2003) National and regional rates and variation of cervical discectomy with and without anterior fusion, 1990-1999. Spine (Phila Pa 1976) 28:931-940; discussion 940

(3)Cowan JA, Dimick JB, Wainess R, Upchurch GR, Chandler WF, La Marca F (2006) Changes in the utilization of spinal fusion in the United States. Neurosurgery 58:15-19

(4)Davis H (1994) Increasing rates of cervical and lumbar spine surgery in the United States, 1979-1990. Spine (Phila Pa 1976) 19:1117-1124

(5)Einstadter D, Kent DL, Fihn SD, Deyo RA (1993) Variation in the rate of cervical spine surgery in Washington State. Med Care 31:711-718

(6)Kristiansen J, Balteskard L, Slettebø H, Nygaard ØP, Lied B, Kolstad F, Solberg TK (2016) The use of surgery for cervical degenerative disease in Norway in the period 2008-2014 : A population-based study of 6511 procedures. Acta Neurochir (Wien) 158:969-974

(7)Lad SP, Patil CG, Berta S, Santarelli JG, Ho C, Boakye M (2009) National trends in spinal fusion for cervical spondylotic myelopathy. Surg Neurol 71:66-69; discussion 69

(8)Liu CY, Zygourakis CC, Yoon S, Kliot T, Moriates C, Ratliff J, Dudley RA, Gonzales R, Mummaneni PV, Ames CP (2017) Trends in Utilization and Cost of Cervical Spine Surgery Using the National Inpatient Sample Database, 2001 to 2013. Spine 42:E906-E913

(9)Marawar S, Girardi FP, Sama AA, Ma Y, Gaber-Baylis LK, Besculides MC, Memtsoudis SG (2010) National trends in anterior cervical fusion procedures. Spine 35:1454-1459

(10)Marquez-Lara A, Nandyala SV, Fineberg SJ, Singh K (2014) Current trends in demographics, practice, and in-hospital outcomes in cervical spine surgery: a national database analysis between 2002 and 2011. Spine (Phila Pa 1976) 39:476-481

(11)Oglesby M, Fineberg SJ, Patel AA, Pelton MA, Singh K (2013) Epidemiological trends in cervical spine surgery for degenerative diseases between 2002 and 2009. Spine (Phila Pa 1976) 38:1226-1232

(12)Patil PG, Turner DA, Pietrobon R (2005) National trends in surgical procedures for degenerative cervical spine disease: 1990-2000. Neurosurgery 57:753-758

(13)Salzmann SN, Derman PB, Lampe LP, Kueper J, Pan TJ, Yang J, Shue J, Girardi FP, Lyman S, Hughes AP (2018) Cervical Spinal Fusion: 16-Year Trends in Epidemiology, Indications, and In-Hospital Outcomes by Surgical Approach. World Neurosurg. doi: 10.1016/j.wneu.2018.02.004

(14)Stein BE, Hassanzadeh H, Jain A, Lemma MA, Cohen DB, Kebaish KM (2014) Changing trends in cervical spine fusions in patients with rheumatoid arthritis. Spine (Phila Pa 1976) 39:1178-1182

(15)Wang MC, Kreuter W, Wolfla CE, Maiman DJ, Deyo RA (2009) Trends and variations in cervical spine surgery in the United States: Medicare beneficiaries, 1992 to 2005. Spine (Phila Pa 1976) 34:955-961

(16)Zeidman SM, Ducker TB, Raycroft J (1997) Trends and complications in cervical spine surgery: 1989-1993. J Spinal Disord 10:523-526

Supplementary Table S2. The WHO ICD-9[a]- and the corresponding ICD-10[b]-codes used to identify and exclude patients with a previous cervical spine operation between and including 1987 and 1998.

WHO ICD-9	WHO ICD-10	Definition
7200A<br>7140A	M05.8	Seropositive rheumatoid arthritis
7209X<br>7140B	M06.0	Other rheumatoid arthritis
7210A	M47.8<br>M47.9	Other spondylosis without myelopathy or radiculopathy<br>Unspecified spondylosis
7211A	M47.1<br>M48.0	Spondylosis with myelopathy
7220A		Cervical disc prolapse without myelo- or radiculopathy
7224A	M50.3	Other cervical disc disorder
7224B		Intervertebral disc degeneration with instability
7227A	M50.0(*G99.2)<br>M50.1<br>G55.1	Cervical disc disease with myelo- or radiculopathy
7228A	M96.1	Cervical post-laminectomy syndrome
7230A	M48.0	Cervical spinal canal stenosis
7230B	M43.3<br>M43.4	Atlanto-axial subluxation

[a] *WHO ICD-9/* [b]*-10* World Health Organization International Classification of Diseases 9th / 10th revision

Supplementary Table S3. The WHO ICD-9[a] and ICD-10[b], SII[c] special medicine reimbursement rights codes and ATC[d]-codes for prescription medicines used to record the comorbidities.

Comorbidity	ICD-9	ICD-10	SII special reimbursement code	ATC
Rheumatoid arthritis	710*, 714*, 725*, 7200*	M05*, M06*, M45*	202	
Hypertension	40*	I10*-I15*	205	C03*, C07*, C09A*, C09B*, C09C*, C09D*, C08*
Atrial fibrillation	4273	I48	207	B01AA03
Cardiac insufficiency	428*	I50*	201	
Coronary artery disease	401*-414*	I20*-I25*	206, 214, 280	
Peripheral artery disease	440*	I70*		
Dyslipidemia	272*, 75989	E78*	211	C10AA*
Diabetes	250	E10*-E14*	103	A10A*, A10B*
Renal insufficiency	585*	N18*	137	
Cancer	140*-208*	C00*-C99*, D00*-D09*	115, 116, 117, 128, 130, 180, 184, 185, 189, 311, 312, 316	L01* (excluding L01BA01)
Chronic obstructive pulmonary disease or asthma	4912*, 496*, 493*	J44*-J46*	203	R03*
Dementia	290*, 3310*	F00*-F03*, G30*	307	N06D*
Demyelinating disease or other degenerative disease of the CNS[e]	3312*-3319*, 333*-336*, 340*	G10*-G13*, G31*, G35*, G36*, G37*	109	
Polyneuropathy	356*, 357*	G60*, G61*, G62*, G63*		
Mononeuropathies	353*, 354*, 355*	G54*, G56*, G57, G58, G59		
Parkinson's disease	332*	G20*	110	N04B*
Epilepsy	345*	G40*, G41*	111	N03A*
Cerebrovascular disease (incl. haemorrhagic and ischemic stroke and TIA[f])	430*-483*	G45*, I60*-I66*, I68*, I69*, G46* with mention of G45, I60*-I69*		

Depression	2960*, 2961*	F32*-F34*		N06A*
Other mental disorder	295*-298* (excluding 2960* and 2961*)	F20*-F31*	112, 118	N05A* (excluding N05AB01 and N05AB01 and no dementia)
Alcohol/drug addiction	291*, 304*, 305*	F10*-F19*		
Operated coxarthrosis / gonarthrosis	71515, 71525, 71735, 71595 or 71516, 71526, 71536, 71595	F16* + NFB* or M17* + NGB*		
Operated shoulder osteoarthrosis	71500, 71509, 71589, 71580, 71590, 71511, 71521, 71531, 71591	M15* (excluding M51.1, M51.2), M19* + NBF*, NBG*		
Rotator cuff syndrome	72610, 72613, 72761	M75.1		
Fibromyalgia	7291	M79.7		

[a]*WHO- ICD-9/*[b]*-10* World Health Organization International Classification of Diseases 9th/10th revision, [c]*SII* Social Insurance Institute of Finland, [d]*ATC* Anatomical Therapeutic Chemical, [e]*CNS* Central nervous system, [f]*TIA* Transient ischemic attack

Supplementary Table S4. Catchment population, degenerative cervical spine patient demographics and number of neurosurgery specialists by year, 1999-2015.

	Catchment population (age 18 or older)			Operated patients			Neurosurgery specialists	
**Year**	**Population**	**Mean age (y)**	**Female (%)**	**N**	**Mean age (y±SD)**	**Female (%)**	**N**	**Neurosurgeon frequency***
1999	4005817	47.2	51.9	832	52.5±11.9	43.3	45	1.1 / 100 000
2000	4025642	47.3	51.8	945	51.2±11.4	43.2	47	1.2 / 100 000
2001	4049800	47.5	51.8	779	52.0±11.9	43.5	48	1.2 / 100 000
2002	4070696	47.7	51.7	899	52.1±10.5	39.2	52	1.3 / 100 000
2003	4090381	47.9	51.7	1002	52.1±11.0	41.0	53	1.3 / 100 000
2004	4103145	48.1	51.7	900	52.7±11.2	43.7	56	1.4 / 100 000
2005	4130831	48.3	51.6	1117	52.9±10.9	44.1	59	1.4 / 100 000
2006	4156013	48.5	51.6	1028	53.8±11.5	44.9	59	1.4 / 100 000
2007	4182978	48.7	51.5	1124	53.7±11.2	44.1	63	1.5 / 100 000
2008	4212984	48.8	51.5	1245	52.8±11.1	44.9	70	1.7 / 100 000
2009	4240914	49.0	51.5	1251	53.5±11.0	46.8	73	1.7 / 100 000
2010	4268611	49.1	51.4	1281	53.3±11.1	46.1	75	1.8 / 100 000
2011	4296816	49.2	51.4	1453	54.1±11.3	44.8	78	1.8 / 100 000
2012	4325139	49.4	51.3	1466	53.7±11.4	46.9	81	1.9 / 100 000
2013	4351593	49.5	51.3	1576	53.8±11.7	44.7	82	1.9 / 100 000
2014	4373053	49.7	51.3	1433	54.8±11.8	46.4	84	1.9 / 100 000
2015	4390948	49.9	51.2	1370	54.3±11.8	44.3	92	2.1 / 100 000
**All**	**71275361**	**48.6**	**51.5**	**19701**	**53.3±11.4**	**44.4**	-	-

*Frequency of neurosurgery specialists per 100 000 people in catchment population

Supplementary Figure S5. The annual incidences of operations in the tertiary hospital districts within each university hospital catchment area: Helsinki (a), Kuopio (b), Oulu (c), Tampere (d) and Turku (e).

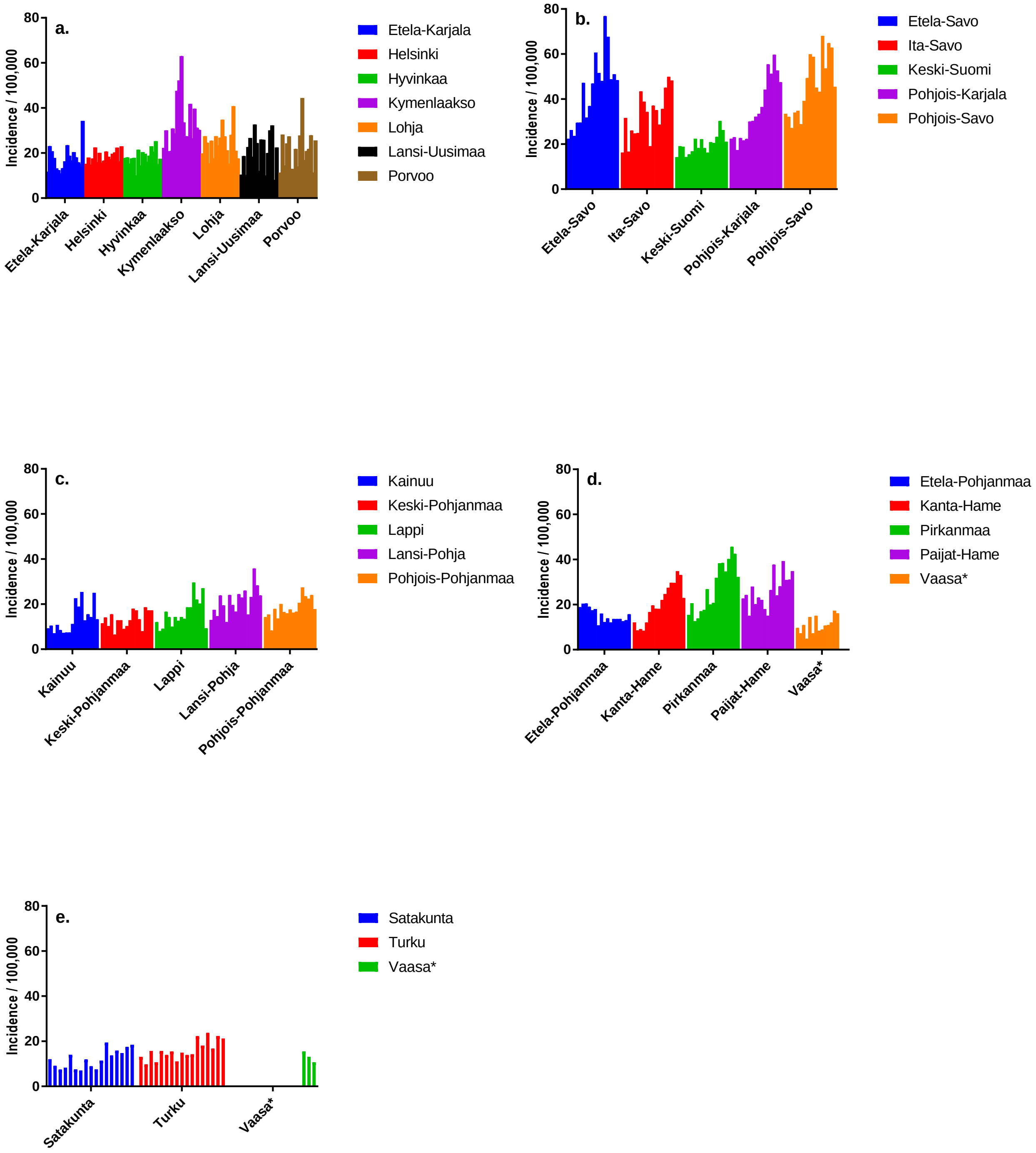

*The hospital district of Vaasa changed the referral university hospital from Tampere to Turku from the year 2013 onward.
